# Identification of a gene expression signature associated with brain metastasis in colorectal cancer

**DOI:** 10.1007/s12094-024-03408-5

**Published:** 2024-03-17

**Authors:** Marlies Michl, Francesco Taverna, Christine Woischke, Pan Li, Frederick Klauschen, Thomas Kirchner, Volker Heinemann, Michael von Bergwelt-Baildon, Arndt Stahler, Tobias Marcus Herold, Vindi Jurinovic, Jutta Engel, Jörg Kumbrink, Jens Neumann

**Affiliations:** 1 Department of Medicine III, University Hospital, Ludwig-Maximilian-University of Munich, Munich, Germany; 2 Department of Haematology and Oncology, Comprehensive Cancer Center Munich, Ludwig-Maximilian-University of Munich, Munich, Germany; 3 Institute of Pathology, Faculty of Medicine, Ludwig-Maximilian-University of Munich, Munich, Germany; 4 German Cancer Consortium (DKTK), Partner Site Munich and German Cancer Research Centre (DKFZ), Heidelberg, Germany; 5 Department of Hematology, Oncology, and Tumorimmunology, Corporate Member of Freie Universitaet Berlin and Humbolt-Universitaet zu Berlin, Charité – Universitaetsmedizin Berlin, Berlin, Germany; 6 Institute for Medical Information Processing, Biometry and Epidemiology, Ludwig-Maximilian-University of Munich, Munich, Germany; 7 Munich Cancer Registry (MCR), Ludwig-Maximilian-University of Munich, Munich, Germany

**Keywords:** Colorectal cancer, Brain metastasis, Gene expression signature, Metastatic organotropism

## Abstract

**Purpose:**

Brain metastasis (BM) in colorectal cancer (CRC) is a rare event with poor prognosis. Apart from (K)RAS status and lung and bone metastasis no biomarkers exist to identify patients at risk. This study aimed to identify a gene expression signature associated with colorectal BM.

**Methods:**

Three patient groups were formed: 1. CRC with brain metastasis (BRA), 2. exclusive liver metastasis (HEP) and, 3. non-metastatic disease (M0). RNA was extracted from primary tumors and mRNA expression was measured using a NanoString Panel (770 genes). Expression was confirmed by qPCR in a validation cohort. Statistical analyses including multivariate logistic regression followed by receiver operating characteristic (ROC) analysis were performed.

**Results:**

EMILIN3, MTA1, SV2B, TMPRSS6, ACVR1C, NFAT5 and SMC3 were differentially expressed in BRA and HEP/M0 groups. In the validation cohort, differential NFAT5, ACVR1C and SMC3 expressions were confirmed. BRA patients showed highest NFAT5 levels compared to HEP/M0 groups (global *p* = 0.02). High ACVR1C expression was observed more frequently in the BRA group (42.9%) than in HEP (0%) and M0 (7.1%) groups (global *p* = 0.01). High SMC3 expressions were only detectable in the BRA group (global *p* = 0.003). Only patients with BM showed a combined high expression of NFAT5, ACVR1C or SMC3 as well as of all three genes. ROC analysis revealed a good prediction of brain metastasis by the three genes (area under the curve (AUC)  = 0.78).

**Conclusions:**

The NFAT5, ACVR1C and SMC3 gene expression signature is associated with colorectal BM. Future studies should further investigate the importance of this biomarker signature.

**Supplementary Information:**

The online version contains supplementary material available at 10.1007/s12094-024-03408-5.

## Introduction

Although significant progress has been made in cancer medicine understanding of organ specific metastasis development remains limited. However, knowledge on molecular mechanisms of organotropic metastasis is essential for biomarker-based prediction and prognosis, invention of innovative therapeutic strategies, and consequently improvement of patient outcomes [1]. The term `metastatic organotropism´ describes the distribution of distant metastases to certain organs in a non-random process which is regulated by multiple factors such as subtypes of cancer, molecular features of cancer cells, host immune microenvironment, as well as cross-talk and interactions with local cells [1]. 

To date, formation of brain metastasis (BM) from colorectal carcinoma (CRC) is virtually not understood. Compared to other solid tumors, colorectal BM is less common and a rare event. Incidence rates are reported between one and four percent, however, increasing numbers are observed in the last decades [2–4]. Thus far, only two independent factors exist to predict the development of BM in CRC, namely first, the presence of lung or bone metastasis and second the presence of a (K)RAS mutation [5–7]. Patients with KRAS mutated CRC carry a 3.7-fold higher risk to develop BM during their course of disease [8]. Primary tumor site was recently shown to have no predictive impact [4]. When BM is diagnosed prognosis is utterly devastating with survival times of only few months [9, 10]. Thus, intense efforts for a better understanding of pathogenetic mechanisms regarding the formation of colorectal BM as well as the characterization and identification of patients at risk is urgently needed [11].

Biological findings accumulate that corroborate the hypothesis of organotropism implicating that different molecularly codified organotropic CRC-types determine the metastatic pattern early in disease [12–15]. Thus, intensified investigations regarding pathogenetic mechanisms of colorectal BM development, molecular pathological profiles and debates on how best to identify CRC patients at risk are justified.

Earlier we showed that—in contrast to liver metastasis—other mechanisms than deregulation of Wnt/β-catenin-signaling and acquisition of cancer stemness are required for formation of BM [14]. In contrast, the hypothesis of stem cell driven brain metastatic genesis in CRC was strengthened by the detection of stem cell properties in human brain metastasis stem cell lines.

The present work aimed to identify a gene expression signature associated with BM in CRC. For this purpose, a case control study population was designed consisting of three CRC patient cohorts with different organotropic metastatic phenotypes. Gene expression profiling on mRNA level was performed in primary tumor tissue specimen and compared between groups. Validation of identified markers was carried out applying reverse transcriptase (RT) qPCR expression analysis.

## Materials and methods

### Patients

**Fig. 1:**
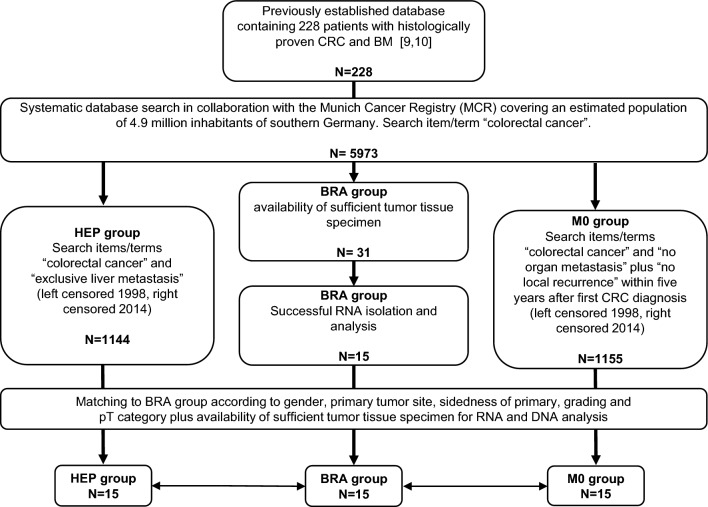
Consort diagram. Illustration of patient selection for the present analysis

All patients involved in the present analysis had a histologically proven diagnosis of CRC with either brain metastasis (BM) or exclusive liver metastasis (HEP) or non-metastatic disease for at least five years (M0) and were diagnosed at the Institute of Pathology, Faculty of Medicine, Ludwig-Maximilians-University (LMU) Munich. Suitable participants for the BM group were selected from the previously built database containing 228 patients with CRC and BM [10, 16] by taking into account the availability of sufficient tumor tissue specimens for the planned expression analyses. The matching HEP and M0 groups were identified via a systematic database search in collaboration with the Munich Cancer Registry (MCR). The MCR covered an estimated population of 4.9 million inhabitants in southern Germany. Proceedings on patient selection for the present study are illustrated in Fig. 1 Proceedings on patient selection for matched-pair analysis were described by our group earlier [14, 15].

### Study design

The present investigation consisted of a matched-pair analysis where patients from all three groups were matched according to gender, primary tumor site (colon versus rectum), sidedness of primary (right versus left colon), grading and pT-category where applicable. As suitable for a matched-pair analysis, all groups consisted of equal patient numbers. Availability of sufficient tumor tissue specimen limited patient numbers to 15 patients per group.

### Histopathological samples

Histopathological diagnosis and classification were reviewed for every available tumor specimen at the accredited Institute of Pathology of the University of Munich (Germany). Histopathological grade was confirmed by an experienced pathologist.

### RNA extraction from FFPE samples

Sections from formalin fixed paraffin embedded (FFPE) tissue samples were prepared followed by hematoxylin–eosin staining of one slide. Areas with a minimum percentage of 50% tumor cells were microdissected from subsequent unstained sections and used for RNA preparation. Total RNA was extracted from six to 12 sections of FFPE tissue sections using the RNeasy FFPE Kit (Qiagen, Hilden, Germany) according to the manufacturer’s instructions. RNA yield and purity were assessed using the NanoDrop ND-1000 spectrophotometer (NanoDrop Technologies, Rockland, USA).

### NanoString® nCounter expression analysis

mRNA expression was measured with the NanoString nCounter FLEX Analysis System (NanoString Technologies, Seattle, USA) using 100 ng of total RNA and the PanCancer Progression Panel (770 genes). The Nanostring expression analysis was performed as described previously [12, 17]. Briefly, the nCounter CodeSet was hybridized to 100 ng total RNA for 18 h at 65 °C. Quality control and normalization of the expression data was performed using the default nSolver v4.0 software settings by utilizing reference genes, positive/negative controls, total counts, and binding densities in each sample. Unsupervised hierarchical clustering and principal component analysis (PCA) were performed with the ClustVis web tool [18]. Default settings were used except for heatmap clustering distance for rows and columns, which was set to Euclidean.

### Reverse transcriptase (RT) qPCR expression analysis

Total RNA (25 ng/µl within the reverse transcription reaction) was transcribed into cDNA using Random Hexamer Primer and the RevertAid™ Reverse Transcriptase kit (both Thermo Fisher Scientific, Waltham, USA). NFAT5, ACVR1C and SMC3 expression was analyzed by qPCR using primers and UPL (universal probe library) probes (Roche, Basel, Switzerland) displayed in Table S1 and the LightCycler® 480 Probes Master mix (Roche). qPCRs were analyzed on a Bio-Rad® CFX Connect™ Real-Time PCR Detection System with the Bio-Rad® CFX Manager™ Software 3.1 (Bio-Rad Laboratories, Hercules, USA). GAPDH, YWHAZ and ACTB (β-Actin) were used for normalization of gene of interest (GOI) expression. Similar PCR efficiencies (> 95%) were achieved for all investigated genes. Any analysis with no Cq value or a Cq value above 40 was considered "undetectable" and expression was set to 0.

Relative GOI mRNA expressions were calculated by division of measured Cq values by the average Cq values of GAPDH, YWHAZ and ACTB. The threshold value for high gene expression was determined using ROC (receiver operating characteristic) analysis and Youden's index. The determined cut-offs for high expression were: NFAT5 ≥ 1.87 × 10^−2^, ACVR1C ≥ 6.60 ×  10^−3^, and SMC3 ≥ 13.73 ×   10^−2^.

### Statistical analysis

For comparison of patient and tumor characteristics between groups, a two-sided chi-squared test was used. The global testing of the relative mRNA expression was achieved using the Kruskal–Wallis test and for the head-to-head comparisons of the relative mRNA expression the Mann–Whitney-*U* test was performed. Outliers were selected with Grubbs's test and not considered in the calculations. The significance of correlations of high gene expression and biomarker combinations was calculated using a chi-squared test. Individual risk prediction for brain metastasis was computed by using multivariate logistic regression to obtain coefficients for each gene. Coefficients were multiplied with the continuous expression values for the corresponding gene and subsequently added. To determine how well the risk prediction model discriminates patients with and without brain metastasis ROC analysis was performed. For all statistical tests, SPSS V. 26.0 (IBM Inc., Armonk, NY) or GraphPad PRISM 8 (GraphPad Software, Inc., USA) were employed. A *p*-value lower than 5% (*p* < 0.05) was considered statistically significant. Global p-values define comparisons between all groups.

## Results

### Patient and tumor characteristics

The study population consisted of 45 patients (19 male [42%], 26 female [58%]) with histologically proven adenocarcinoma of the colorectum and brain metastasis (BRA; *N* = 15), exclusive liver metastasis (HEP; *N* = 15) or non-metastatic disease (M0; *N* = 15) as defined above. Baseline patient and tumor characteristics of the analyzed patient cohort are presented in Table 1.

**Table 1:** Patient and tumor baseline characteristics of the entire study population (*N *= 45)

Total *N* = 45	BRA *N* = 15 (%)	HEP *N* = 15 (%)	M0 *N* = 15 (%)	*P*	
				Global	BRA vs. HEP
Sex					
Female	9 (60.0)	7 (46.7)	10 (66.7)	0.53	0.46
Male	6 (40.0)	8 (53.3)	5 (33.3)		
Age at first diagnosis of CRC					
Mean, years	64.1	62.5	73.3	0.004	0.66
≥ 70 years	4 (26.7)	2 (13.3)	11 (73.3)	0.002	0.36
≥ 65 years	7 (46.7)	7 (46.7)	13 (86.7)	0.04	1.00
< 65 years	8 (53.3)	8 (53.3)	2 (13.3)		
Sidedness of primary					
Right colon	6 (40.0)	6 (40.0)	6 (40.0)	1.00	1.00
Left colon	9 (60.0)	9 (60.0)	9 (60.0)		
Primary tumour site					
Colon	9 (60.0)	9 (60.0)	9 (60.0)		
Rectosigmoid	–	–	–	1.00	1.00
Rectum	6 (40.0)	6 (40.0)	6 (40.0)		
Grading					
Low grade (G1, G2)	10 (66.7)	10 (66.7)	9 (60.0)	0.91	1.00
High grade (G3)	5 (33.3)	5 (33.3)	6 (40.0)		
pT stage					
pT1	–	–	–		
pT2	–	–	4 (26.7)		
pT3	11 (73.3)	12 (80.0)	7 (46.7)	0.07	0.59
pT4	3 (20.0)	3 (20.0)	4 (26.7)		
Unknown	1 (6.7)	–	–		
pN status					
pN0	4 (26.7)	4 (26.7)	4 (26.7)		
pN1	3 (20.0)	3 (20.0)	8 (53.3)		
pN2	7 (46.7)	8 (53.3)	3 (20.0)	0.25	0.79
Unknown	1 (6.7)	–	–		

### Identification of a 7-gene expression signature for brain metastatic CRC

**Fig. 2:**
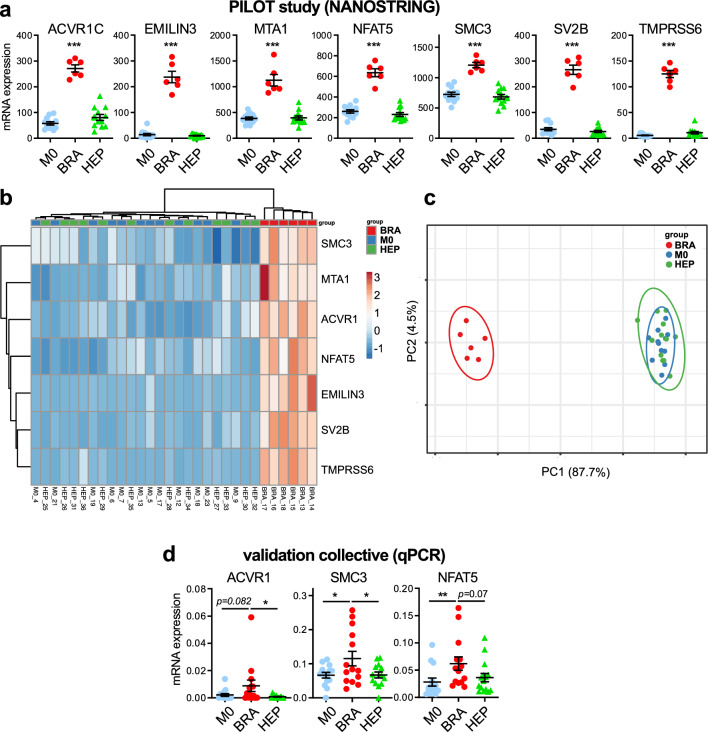
Significantly differentially expressed genes (DEGs) in the M0, BRA and HEP groups. **a** Expression of BRA group associated genes as measured by nanostring analysis in the pilot study cohort (BRA, *n* = 6; M0 and HEP, *n* = 12). **b** Unsupervised hierarchical clustering using the seven DEG. **c** Principal component analysis (PCA) utilizing the seven DEG. **d** Expression of BRA group associated genes as measured in the validation collective (*n* = 15 for each group) by qPCR. **a–d** *BRA* patients with brain metastases; *HEP* patients with liver metastases; *M0* patients without metastases. *, *p *< 0.05. **p < 0.01. ***, *p *< 0.001

In a PILOT study (BRA; *N* = 6; M0 and HEP; *N* = 12 each), analysis of mRNA expression of 770 genes performed with the Nanostring PanCancer Progression Panel was conducted. Analyses revealed differential gene expressions in the investigated patient groups. Specifically, seven genes namely EMILIN3 (Elastin Microfibril Interfacer 3), MTA1 (Metastasis Associated 1), SV2B (Synaptic Vesicle Glycoprotein 2B), TMPRSS6 (Transmembrane Serine Protease 6), ACVR1C (Activin A Receptor Type 1C), NFAT5 (Nuclear Factor Of Activated T Cells 5) and SMC3 (Structural Maintenance Of Chromosomes 3) were significantly differentially expressed in patients of the BRA group (high expression) and patients from the HEP and M0 group (low expression) (Fig. 2a). Moreover, a perfect separation of the BRA group vs M0 and HEP groups was achieved with the seven gene expressions by unsupervised hierarchical clustering (Fig. 2b) and principal component analysis (PCA, Fig. 2c).

### Validation of the identified gene expression profile with qPCR

To confirm the results of the pilot study, expression of the seven identified differentially expressed genes (DEG) was analyzed by qPCR in a larger validation cohort. NFAT5, ACVR1C and SMC3 expression levels were associated with BM, thus confirming the results of the pilot study. Relative mRNA expressions of NFAT5, ACVR1C and SMC3 are listed in Table 2 and illustrated in Fig. 2d. Patients with BM showed the highest mRNA expression of NFAT5 (BRA = 5.08 × 10 ^−2^ [95% CI 2.65 × 10 ^−2^ –10.03 × 10 ^−2^] compared to the HEP group (HEP = 3.00 × 10 ^−2^  [95% CI 1.32 × 10 ^−2^–4.18 × 10 ^−2^]) and the M0 group (M0 = 1.78 × 10 ^−2^ [95% CI 0.81 × 10 ^−2^ –6.36 × 10 ^−2^ global *p* = 0.02). Expression in each case of the BRA group exceeded the threshold value for high NFAT5 expression (BRA: 100.0%; HEP: 60.0%; M0: 42.9%; global *p* = 0.004). Regarding ACVR1C, patients with BM had a stronger ACVR1C expression (BRA = 2.69 × 10 ^−3^  [95% CI 0–13.74 × 10 ^−3^ ]; HEP = 0.18 × 10 ^−3^  [95% CI 0–1.71 × 10 ^−3^]; M0 = 0.33 × 10 ^−3^  [95% CI 0–4.72 × 10 ^−3^ ]) than patients in the HEP and M0 group nearly reaching the level of significance (global *p* = 0.08). High ACVR1C expression was observed more frequently in the BRA group (42.9%) than in the HEP (0%) and M0 (7.1%) group (global *p* = 0.01). Furthermore, the presence of BM was associated with stronger SMC3 expressions than observed in the HEP and M0 group (BRA = 8.16 × 10 ^−2^ [95% CI 4.65 × 10 ^−2^–21.85 × 10 ^−2^ ]; HEP = 6.63 × 10 ^−2^ [95% CI 4.13 × 10 ^−2^–9.73 × 10 ^−2^ ]; M0 = 6.95 × 10 ^−2^ [95% CI 3.68 × 10 ^−2^ –9.63 × 10 ^−2^ ]; global *p* = 0.34). High SMC3 expression was only detectable in the BRA group (35.7%), whereas no case of the HEP and M0 group (global *p* = 0.003) displayed high SMC3 expression.

**Table 2:** Relative mRNA expression and frequency of high and low mRNA expression of NFAT5, ACVR1C and SMC3 comparing the three patient cohorts

Total *N* = 45	BRA *N* = 15 (95% CI)	HEP *N* = 15 (95% CI)	M0 *N* = 15 (95% CI)	Global *P*
Relative mRNA expression				
*NFAT5*	5.08 × 10^−2^ (2.65 × 10^−2^; 10.03 × 10^−2^)	3.00 × 10^−2^ (1.32 × 10^−2^; 4.18 × 10^−2^)	1.78 × 10^−2^ (0.81 × 10^−2^; 6.36 × 10^−2^)	**0.02**
*ACVR1C*	2.69 × 10^−3^ (0; 13.74 × 10^−3^)	0.18 × 10^−3^ (0; 1.71 × 10^−3^)	0.33 × 10^−3^ (0; 4.72 × 10^−3^)	0.08
*SMC3*	8.16 × 10^−2^ (4.65 × 10^−2^; 21.85 × 10^−2^)	6.63 × 10^−2^ (4.13 × 10^−2^; 9.73 × 10^−2^)	6.95 × 10^−2^ (3.68 × 10^−2^; 9.63 × 10^−2^)	0.34
Frequency of high and low mRNA expression				
* NFAT5* expression	*N* = 14 (%)	*N* = 15 (%)	*N* = 14 (%)	
Low	0 (0)	6 (40.0)	8 (57.1)	**0.004**
High	14 (100)	9 (60.0)	6 (42.9)	
* ACVR1C* expression	*N* = 14 (%)	*N* = 13 (%)	*N* = 14 (%)	
Low	8 (57.1)	13 (100)	13 (92.9)	**0.01**
High	6 (42.9)	0 (0)	1 (7.1)	
*SMC3* expression	*N* = 14 (%)	*N* = 14 (%)	*N* = 14 (%)	
Low	9 (64.3)	14 (100)	14 (100)	**0.003**
High	5 (35.7)	0 (0)	0 (0)	

Significant p-values are printed in bold

### Different gene expression combinations and head-to-head comparisons of the investigated organotropic patient groups

Next, we tested for the association of combined expressions of the identified DEG with certain groups. Patients with BM were characterized by the simultaneous presence of high NFAT5, ACVR1C and/or SMC3 expression. A combined high expression of NFAT5 and ACVR1C (*N* = 5 [38.5%]), NFAT5 and SMC3 (*N* = 5 [35.7%]), ACVR1C and SMC3 (*N* = 3 [23.1%]) as well as of all three genes (*N* = 3 [23.1%]) was observed exclusively in the BRA group (Table 3). No case of the HEP and M0 group expressed one of these combinations, resulting in a significant difference between groups. Head-to-head- expression comparisons between groups (Table 4) showed a significant difference in NFAT5 expression between the BRA and the M0 group (*p* = 0.004) and, in ACVR1C (BRA vs. HEP group; *p* = 0.04). High NFAT5, ACVR1C and SMC3 expression levels differed significantly between the BRA and M0 group (NFAT5 *p* = 0.001; ACVR1C *p* = 0.03; SMC3 *p* = 0.01) and the BRA and HEP group (NFAT5 *p* = 0.01; ACVR1C *p* = 0.01; SMC3 *p* = 0.01), but not between the HEP and M0 group (NFAT5 *p* = 0.36, ACVR1C *p* = 0.33; SMC3 *p* = 1.00). Analyses of combined biomarker expressions revealed only significant differences when comparing high combined NFAT5 and ACVR1C expression (BRA vs. M0, *p* = 0.01; BRA vs. HEP, *p* = 0.01) as well as high NFAT5 and SMC3 expression (BRA vs. M0, *p* = 0.02; BRA vs. HEP, *p* = 0.01).

**Table 3:** Frequency of biomarker combinations comparing the three patient cohorts. Patient numbers are indicated without outliers

Biomarker combinations	BRA		HEP		M0		Global *P*
	Positive	Negative	Positive	Negative	Positive	Negative	
	*N*= 13 (%)		*N*= 13 (%)		*N* = 13 (%)		
*NFAT5* high expression PLUS *ACVR1C* high expression	5 (38.5)	8 (61.5)	0 (0.0)	13 (100)	0 (0)	13 (100)	**0.003**
	*N* = 14 (%)		*N* = 14 (%)		*N* = 13 (%)		
*NFAT5* high expression PLUS *SMC3* high expression	5 (35.7)	9 (64.3)	0 (0.0)	14 (100)	0 (0)	13 (100)	**0.004**
	*N* = 13 (%)		*N* = 12 (%)		*N* = 13 (%)		
*ACVR1C* high expression PLUS *SMC3* high expression	3 (23.1)	10 (76.9)	0 (0)	12 (100)	0 (0)	13 (100)	**0.04**
	*N* = 13 (%)		*N* = 12 (%)		*N* = 12 (%)		
*NFAT5* high expression PLUS *ACVR1C* high expression PLUS *SMC3* high expression	3 (23.1)	10 (76.9)	0 (0)	12 (100)	0 (0)	12 (100)	**0.049**

Confidence intervals (CI) are shown in brackets

Significant p-values are printed in bold

**Table 4:** Head-to-head comparisons between groups according to mRNA expression and biomarker combinations

	BRA ↔M0	HEP ↔ M0	BRA ↔ HEP
Relative mRNA expression			
* NFAT5*	**0.004**	0.29	0.07
* ACVR1C*	0.08	0.91	**0.04**
* SMC3*	0.21	0.92	0.23
High mRNA expression			
* NFAT5* high expression	**0.001**	0.36	**0.01**
* ACVR1C* high expression	**0.03**	0.33	**0.01**
* SMC3* high expression	**0.01**	1.00	**0.01**
Biomarker combinations			
*NFAT5* high expression PLUS *ACVR1C* high expression	**0.01**	1.00	**0.01**
*NFAT5* high expression PLUS *SMC3* high expression	**0.02**	1.00	**0.01**
*ACVR1C* high expression PLUS *SMC3* high expression	0.07	1.00	0.08
*NFAT5* high expression PLUS *ACVR1C* high expression PLUS *SMC3* high expression	0.08	1.00	0.08

Significant p-values are printed in bold

To test whether the three DEG signature can be used to predict the risk of BM, a multivariate logistic regression and subsequent ROC analysis was applied. An area under the curve (AUC) of 0.78 was achieved, even though patient numbers were low (Supplementary Fig. 1), suggesting an acceptable discrimination between patients that will develop brain metastases and those who will not.

## Discussion

The present work aimed to identify a gene expression signature predictive for the development of colorectal brain metastasis. Brain metastasis from CRC represents a rare event, but numbers are increasing. Still, prognosis for most CRC patients affected is outstandingly poor [10, 16] and thus, there is a high medical need to identify patients at risk. To date, the only known independent factors to predict the development of BM in CRC are the presence of lung or bone metastasis as well as the presence of a (K)RAS mutation [5–8].

In this manuscript we report results from a case–control-analysis comparing gene expression profiles of three CRC patient cohorts with different organotropic metastatic phenotypes. The three study groups consisted of (1) CRC patients with brain metastasis (BRA group), (2) CRC patients with exclusive liver metastasis (HEP group) and CRC patients without metastatic disease within five years after CRC diagnosis (M0 group).

For the present study, we deliberately chose an approach from the clinicians perspective and analyzed primary CRC tumor tissue facing the question whether primary CRC tissue provides information on metastatic organotropism with focus on colorectal BM. Therefore, we designed a reverse translational study “from bedside to bench” and formed the above described organotropic patient groups by incorporating a matched-pair technique to make groups as homogenous as possible. To our knowledge, no previous studies on this topic with a comparable study design have been published so far.

In the PILOT study, we identified the seven genes EMILIN3, MTA1, SV2B, TMPRSS6, ACVR1C, NFAT5 and SMC3, showing a significantly higher mRNA expression in the BRA group compared to the HEP and M0 group. Results on high mRNA expression of NFAT5, ACVR1C and SMC3 in patients from the BRA group and low expression of the same three genes in the HEP and M0 group were confirmed in the validation cohort with a second independent analysis method. Moreover, we show that this three gene expression signature might have predictive impact for the formation of colorectal BM as an AUC of 0.78 was achieved even if the collective was small.

The Nuclear Factor of Activated T-cells 5 (NFAT5) was originally identified as tonicity regulated transcription factor and plays a central role in the adaptation of cells to osmotic stress [19]. NFAT5 is upregulated by hyperosmolarity caused by local inflammatory reaction e.g. induced by tumor growth [19]. Thus, strong NFAT5 expression could indicate an activated immune response that curtails tumor aggressiveness and consequently tapers down the tempo of metastatic spread. As known, mCRC patients with BM often show longer courses of disease compared to mCRC patients with liver and/or peritoneal metastasis suggesting less aggressive tumor biology or potentially a better immunologic tumor control [15, 20, 21].

The Activin A Receptor Type IC (ACVR1C) also known as ALK7 and its ligand nodal growth differentiation factor (NODAL), is a type I receptor serine/threonine kinase to which TGF-β ligands bind [22]. By activating the subsequent signaling pathway cell proliferation is reduced [23, 24]. In a cancer stem cells enriched colorectal cancer spheroid cell model, ACVR1C was described as one of the six key molecules involved in signaling pathways for controlling various aspects of cancer stem cells [25]. Increased expression of ACVR1C seems to be associated with a less aggressive tumor growth and high ACVR1C expression was a positive prognostic factor in several tumor entities [26].

The Structural Maintenance of Chromosomes 3 (SMC3) is a member of the SMC protein family and a key regulator of DNA repair, chromosome condensation and chromosome segregation [27, 28]. Since SMC3 may also influence the activation of β-catenin [29] and this in turn can lead to EMT [30], SMC3 may directly activate metastatic growth.

Certainly, there are several limitations of the present investigation. First, low patient and tumor numbers limit meaningfulness of the presented results and merely grant this data a hypothesis-generating impact. Second, the retrospective and explorative study design implicates compromises in quality and completeness of available data on e.g. patients and tumor characteristics. However, we are convinced that for rare cancers which certainly include brain metastatic CRC acquisition of tumor tissue and data from prospective trials is not realistic.

In conclusion, the presented work identified the seven genes EMILIN3, MTA1, SV2B, TMPRSS6, ACVR1C, NFAT5 and SMC3 associated with the formation of colorectal BM during the course of disease but not with liver metastasis or non-metastatic disease. High mRNA expression of the three genes NFAT5, ACVR1C and SMC3 was confirmed with a second validation analysis technique. We suggest that primary colorectal tumors apparently contain gene expression markers which precede the formation of BM. Even if the present study cohort is small and prone to bias, considerations on carrying out such analyzes systematically are justified. Further, underlying mechanisms need to be validated in larger study cohorts and functional experiments.

## Supplementary Information

Below is the link to the electronic supplementary material.

Supplementary file1 (PDF 47 KB)

Supplementary file1 (Docx 15 KB)

## Data Availability

The datasets used and/or analyzed during the current study are available from the corresponding author on reasonable request.
