# Supplementary figures and images for: Identification of a gene expression signature associated with brain metastasis in colorectal cancer
Source: Clin Transl Oncol. 2024 Mar 17;26(8):1886–95. doi: 10.1007/s12094-024-03408-5 (PMC11249597; doi:10.1007/s12094-024-03408-5)

individual risk prediction

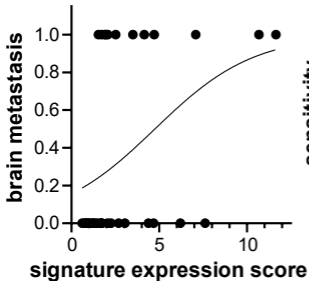

ROC curve

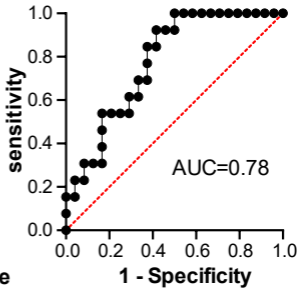

Supplement: Supplementary file 1 — Supplementary file1 (PDF 47 KB) [file 12094_2024_3408_MOESM1_ESM.pdf]
