# Supplementary material for: Identification of a gene expression signature associated with brain metastasis in colorectal cancer
Source: Clin Transl Oncol. 2024 Mar 17;26(8):1886–95. doi: 10.1007/s12094-024-03408-5 (PMC11249597; doi:10.1007/s12094-024-03408-5)
Supplement: Supplementary file 2 — Supplementary file1 (Docx 15 KB) [file 12094_2024_3408_MOESM2_ESM.docx]

Table S1 Primer sequences and detection probes

| **Gene** | **Forward (5‘−3‘)** | **Reverse (5‘−3‘)** | **Pupose** | **UPL-#** |
| --- | --- | --- | --- | --- |
| *ACTB* | ACAGGATGCAGAAGGAGATCA | CGATCCACACGGAGTACTTG | Expression normalization | 63 |
| *ACVR1C* | TTATGATGTGACCGCCTCTG | TGCAATTGTCCTTTGAACCA | mRNA expression | 21 |
| *GAPDH* | AGCCACATCGCTCAGACAC | GCCCAATACGACCAAATCC | Expression normalization | 60 |
| *NFAT5* | GTCAGACAAGCGGTGGTGA | TGCTTCAGAGGCAAATCCA | mRNA expression | 25 |
| *SMC3* | TGGAGAAAAATCCAGACAATTAAGA | ACTTGGCGTTCGATATCCTC | mRNA expression | 18 |
| *YWHAZ* | GATCCCCAATGCTTCACAAG | TGCTTGTTGTGACTGATCGAC | Expression normalization | 30 |
